# Preventing Late-Life Depression: Lessons in Intervention Development From Goa, India

**DOI:** 10.1093/geroni/igx030

**Published:** 2018-01-24

**Authors:** Charles F Reynolds, Amit Dias, Alex Cohen, Jennifer Morse, Stewart J Anderson, Pim Cuijpers, Vikram Patel

**Affiliations:** 1 School of Medicine, University of Pittsburgh, Pennsylvania; 2 Graduate School of Public Health, University of Pittsburgh, Pennsylvania; 3 Goa Medical College, Goa, India; 4 Sangath, Goa, India; 5 London School of Hygiene and Tropical Medicine, UK; 6 Chatham University, Pittsburgh, Pennsylvania; 7 Department of Psychology, Free University of Amsterdam, the Netherlands; 8 Harvard Medical School, Boston, Massachusetts

**Keywords:** Depression and anxiety prevention, Primary care, Stress and coping

## Abstract

We describe the development of an intervention strategy for the indicated prevention of depression in older adults living in Goa, India. Of particular novelty, the intervention is deliverable by lay health counselors and is grounded in problem solving therapy for primary care and brief behavioral treatment for insomnia. We have named the intervention “DIL” (the Hindi word for “heart” and an acronym for “depression in late life.”) Additional DIL strategies include psychoeducation in self-management of co-occurring medical disorders such as diabetes mellitus, together with assistance in navigation to needed social and economic resources. We present the results of a preliminary open-trial case series involving 21 participants with subsyndromal symptoms of depression, demonstrating feasibility, acceptability, and benefit to participants. We then present the design of a larger confirmatory trial into which 181 participants have been enrolled. “DIL” is a novel and large depression prevention trial conducted with lay health counselors in a low-resource country. Its results are likely to have implications for depression prevention in older adults in other low- and middle-income countries and to inform contemporary models of the staging of depressive illness in later life.

**Translational Significance**

The training of lay health counselors to deliver simple behavioral interventions such as problem solving therapy and brief behavioral treatment for insomnia, combined with education in the self-management of common co-occurring medical disorders and navigational assistance to social services, may prove to be a pragmatic and scalable approach to reducing incidence of clinical depression among at-risk older adults in low-resource settings. Preventing depression may also repay down-stream benefits of prolonged health span and independence by promoting cognitive health in the later years of life.

## Overview

Prevention of depressive and anxiety disorders is of great public health significance in low- and middle-income countries (LMIC), and represents one of the grand challenges in global mental health (grandchallengesinglobalMH@NIH.org). Late-life depression and anxiety disorders, which frequently co-exist, are of particular concern in LMIC due to rapid demographic transition and aging in countries such as India; increased prevalence of social conditions that are recognized as risk factors (eg, living alone or living with a chronic disabling condition); and the weak response of health systems to address the needs (let alone the mental health needs) of the elderly. Because of the lack of mental health specialists, there is a need to focus on preventive interventions that can be delivered by nonspecialists and lay health counselors (LHCs) in nonhealth care or primary care settings (1). The ongoing work described here also has relevance to policy and practice in high-income countries by clarifying appropriate roles for lay- and nonspecialist workers in depression and anxiety prevention for populations with few mental health resources.

Meta-analyses of more than 30 randomized trials in high-income countries show that the incidence of new and recurrent episodes of major depression and commonly co-occurring anxiety disorders can be reduced by 20%–25% over 1–2 years compared to usual care (2–4). The MANAS trial (“project to promote mental health”) conducted in Goa, India demonstrated that the use of LHCs, as part of a collaborative stepped-care intervention, increased recovery rates from common mental disorders (anxiety and depression) in a mixed-age sample of patients attending public primary care clinics (5). An unanticipated finding was that the MANAS intervention also reduced the incidence of common mental disorders in patients with initially subthreshold (subsyndromal) depressive and anxiety symptoms. Given the shortage of mental health specialists in LMIC, MANAS successfully demonstrated the feasibility and importance of task sharing and task shifting, that is, the rational re-distribution of tasks among health workforce teams to make more efficient use of lay and professional human resources. The work reported here investigates the use of nonspecialist LHCs in the effort to prevent late-life depression and anxiety in LMIC. DIL aims to test the value of relatively low-intensity psychosocial interventions for depression prevention, based upon observations reported in the MANAS trial.

We have followed the Medical Research Council Guidelines in formative research for the development of complex interventions, to create and standardize a preventive intervention for use by LHCs in primary care clinics in Goa, India. We have named our model “DIL” (an acronym for “depression in later life” and the word for “heart” in Hindi, the national language of India). Using an uncontrolled case series enrolling 21 participants, we conducted an initial test of the feasibility and acceptability of the DIL model. As now developed, DIL comprises psychoeducational interventions delivered by LHCs and previously shown to have preventive efficacy: (a) education about symptoms of depression and anxiety; (b) scheduling of activities, including pleasurable activities, to manage symptoms of depression; and (c) provision of social case work to help older adults navigate to needed resources. Based upon a review of the global depression prevention literature, these psychoeducational strategies are grounded within two approaches that appear to work by strengthening protective factors: problem solving therapy for primary care (6) and brief behavioral treatment for insomnia (7).

Following the completion of formative research, we are now performing a randomized clinical trial of indicated prevention enrolling 181 older primary care participants with subsyndromal symptoms of depression and anxiety. This has allowed us to gather data on the feasibility of identifying, enrolling, randomizing, and retaining participants; implementing the experimental intervention (“DIL”) and control arm of enhanced usual care under the real-world conditions of public, primary care clinics in Goa; and assessing the fidelity of the DIL implementation.

The work described here has been supported by an NIMH intervention development grant (R34 MH 96997) to the University of Pittsburgh and carried out in partnership with Sangath, an NGO in Goa that has conducted scalable mental health research across the life cycle for over 20 years. Additional partners are the London School of Hygiene and Tropical Medicine, the Goa Medical College, the Free University of Amsterdam, and Harvard Medical School. We have registered “DIL” at clinicaltrials.gov.

## Significance and Innovation

Why is prevention of major depression in later life important, especially in LMIC? The public health case for depression prevention in older adults rests upon the following factors:

(1) The number of older adults in LMICs, including India, will grow substantially in the next few decades.
(2a) *Prevalence and course of major depression*: Episodes of major depression in older adults are prevalent and disabling (6%–10% in primary care settings; 30% in inpatient and long-term care settings).
(2b) The disorder often runs a relapsing and chronic course, and social factors related to economic and social disadvantages (low education and violence) are major determinants.
(2c) Major depression often co-occurs with other chronic conditions like diabetes and hypertension, amplifying the disability associated with these conditions, undermining treatment adherence and worsening family caregiver burden.
(2d) Depression is associated with excess mortality after myocardial infarction, stroke, and cancer and is the major risk factor stroke in old age.
(3) *Available treatments*: Current treatments are only partially satisfactory in reducing symptom burden, sustaining remission, and averting years lived with disability.
(4a) *Problems for treatment of depression inLMIC*: The treatment gap for people with mental disorders has been extensively documented, especially in LMICs where up to 90% of affected persons do not receive treatment.
(4b) The scarcity of mental health specialists in most countries and the inequity of the distribution of these specialists is a major barrier to closing the treatment gap. The existence of the treatment gap and the attendant workforce issues underscore the need for developing effective models of prevention that can be implemented by health workers with shorter training and fewer qualifications, to optimize the efficient use of the available human resources for health. Finally, mental health practices targeting the prevention, early detection, and treatment of depression across the lifespan (particularly later in life) could contribute to the reduction in the incidence and prevalence of dementing disorders (8).

## Evidence-Based Approaches Circa 2017

### Prevention

If one conceptualizes a staging model of depressive illness, analogous to the stages of cancer, then prevention, or early intervention, in persons with subsyndromal symptoms (the essence of what the Institute of Medicine has termed “indicated” prevention) may represent an opportunity to delay, mitigate, or altogether prevent the development of a later stage of illness, namely, the syndromal expression of major depression and its attendant risk for relapse, recurrence, and other down-stream consequences, including dementia and suicide. Just as in oncology early intervention targets more treatable stages of illness with better prognosis, so also with respect to depression, early intervention may prevent the emergence of more treatment-resistant illness with worse prognosis. In LMIC, where mental illness treatment resources are scarce, the argument for prevention is all the more compelling for this reason. A staging model of depression, as posited here, represents a useful hybrid of traditional categorical or binomial approaches to diagnosis and of dimensional models representing a continuum of severity. A staging model offers the possibility of different platforms for interventions, prevention- and treatment-oriented, depending upon the stage of illness.

### Impact

Both randomized prevention trials and epidemiological modeling suggest that prevention of major depression in later life may be most efficiently accomplished by targeting elderly persons who experience risk factors, particularly functional limitations as a result of illnesses such as stroke or macular degeneration, have a small social network, and/or have subsyndromal symptoms (9–12). Efficiency of interventions to prevent depression encompasses both the impact of the intervention and the effort required to implement it.

Impact is reflected by the proportion of cases that would be prevented if the adverse effects of targeted risk factors were completely blocked (attributable fraction). Effort is reflected in the number of persons who would need to receive a depression prevention intervention to avoid one new case of late-life depression (number needed to treat [NNT]). Investigators have estimated that preventive interventions would have the highest impact and lowest effort in the presence of subsyndromal depressive symptoms (NNT = 3–4 in “indicated” prevention versus 7–9 in “selective” prevention with at-risk but asymptomatic persons) (13). That is, preventive interventions may have more impact in older adults who already have subthreshold symptoms (“indicated” prevention) than in persons without such symptoms but with disabilities from co-occurring medical and neurologic illness (“selective” prevention). In the work described here, we track both depressive and anxiety symptoms as well as psychosocial stressors (such as family conflict), because persons with subsyndromal symptoms frequently endorse multiple stressors.

### Examples

A study conducted in Amsterdam by van’t-Veer Tazelaar and colleagues (12) evaluated indicated prevention in Dutch primary care patients above the age of 75. With the objective of determining the efficacy of an indicated, stepped-care prevention algorithm, 170 patients with subsyndromal symptoms of depression or anxiety were followed. Comprising the intervention algorithm were four sequential steps of increasing effort, each of 3 months duration: watchful waiting, cognitive behavior therapy-based bibliotherapy, problem solving therapy, and referral to primary care if the patient needed antidepressant medication. The incidence of major depressive episodes and anxiety disorders was reduced by half over a 1-year period. Thus, 24% of participants randomly assigned to treatment as usual experienced the onset of major depression or of anxiety disorders, compared with 11% of participants receiving stepped-care for depression prevention (NNT = 4). This stepped-care algorithm was also cost-effective (12).

A study exemplifying selective prevention (10) enrolled 176 patients with recent stroke. Nondepressed patients receiving placebo (*n* = 58) were more likely over the course of 12 months to suffer a major or minor depressive episode than patients receiving low-dose escitalopram (5 mg/day, *n* = 59) or receiving a course of problem solving therapy (*n* = 58). This study reported an NNT of 8.

We conducted a meta-analysis of incidence rate reduction in major depression, pooling data from 32 studies enrolling mixed-aged samples (4). Thirty-two studies qualified for inclusion, comparing brief psychological and/or behavioral interventions with care as usual. We found an incidence rate ratio was 0.79 (95% CI: 0.69–0.91), indicating a 21% decrease in the incidence in prevention groups versus control groups (typically, usual care).

## Problem Solving Therapy and Brief Behavioral Treatment in the DIL Study

Brief learning-based approaches, already shown to have efficacy in the treatment of depressive disorders, offer a promising strategy to develop and test innovative interventions to reduce risk and positively alter trajectory of depressive illness (by intervening at an earlier stage of illness). While antidepressant medications are the most widely used modality for treating prevalent cases of major depression, their use in subsyndromal depression may be ill-advised due to a lack of evidence for efficacy in mild depression, as well as adverse effects in older adults such as hyponatremia, risk for falls, bone de-mineralization, and cataracts (13). Psychological and behavioral interventions may be better for reasons of safety and patient preference. Problem Solving Therapy for Primary Care (PST-PC), in which behavioral activation is an important component, has been used successfully in depression prevention studies (14,15). PST-PC is more easily administered than interpersonal psychotherapy or cognitive behavioral therapy and can be embedded within a clear service model in the real world of primary care (16). Again, our aim has been to extend the observation reported in the MANAS trial that a relatively low-intensity psychosocial intervention may be effective in depression prevention. PST-PC inculcates a positive problem solving orientation and teaches active coping skills to enhance resilience to stress and to diminish the sense of loss of control (feeling trapped or helpless) at the core of depression’s pathogenesis (6). Similarly, because poor sleep is a well-established risk factor for common mental disorders like depression and anxiety, teaching and practicing strategies for better sleep may enhance active coping by diminishing affective reactivity and enhancing cognitive flexibility (ie, the ability to brain-storm possible solutions and to choose from among these a strategy with an optimal cost-benefit ratio) on the part of both care recipients and care givers (7). Thus, the combination of PST-PC and BBTI may be synergistic. In this context, BBTI improves sleep quality and reduces symptoms of depression and anxiety in primary care patients. Both PST-PC and BBTI are learning-based, skills-enhancing interventions that are safe, cheap, deliverable by general medical clinicians and lay-health counselors, and more likely to be acceptable to older adults than antidepressant medication before major depression or an anxiety disorder is diagnosable.

## Summary of Innovation and Potential Impact of “DIL”

DIL is generating the first evidence of a potentially scalable intervention for prevention of common mental disorders in a low- and middle-income country in the midst of rapid demographic transition (1). The emphasis on mental illness prevention via the use of LHCs and the exploration of a combination of synergistic approaches are both innovative. DIL takes the unanticipated prevention results of MANAS (5) into a specific hypothesis-driven study. In a mixed-age sample with subthreshold symptoms, MANAS showed a prevalence of 12.7% of common mental disorders among those receiving collaborative stepped care administered by LHCs versus 25.0% among those receiving enhance usual care. Like DIL, MANAS deployed LHCs into rural and urban primary care clinics, screened for the presence of common mental disorders (DIL screened for the presence of subsyndromal symptoms rather than current major depression), and utilized a collaborative, stepped-care approach including prescription of antidepressant medication by primary care physicians and supervision by a mental health specialist. DIL’s specific focus on older adults in an LMIC (driven not only by demographic transition but also by older adults’ higher exposure to risk factors for depression, such as subsyndromal symptoms, insomnia, illness-related disabilities, physical pain, and social isolation) breaks new ground in reducing the global disease burden of depression. Finally, since learning-based approaches, such as PST-PC and BBTI, are effective for treating prevalent cases of depression, DIL investigates whether these interventions will be acceptable, feasible, safe, and effectively administered by LHCs to preventing depression and anxiety in at-risk older adults in LMIC.

## Research Approach: Experimental Design and Methods Used in DIL

**Figure 1.**
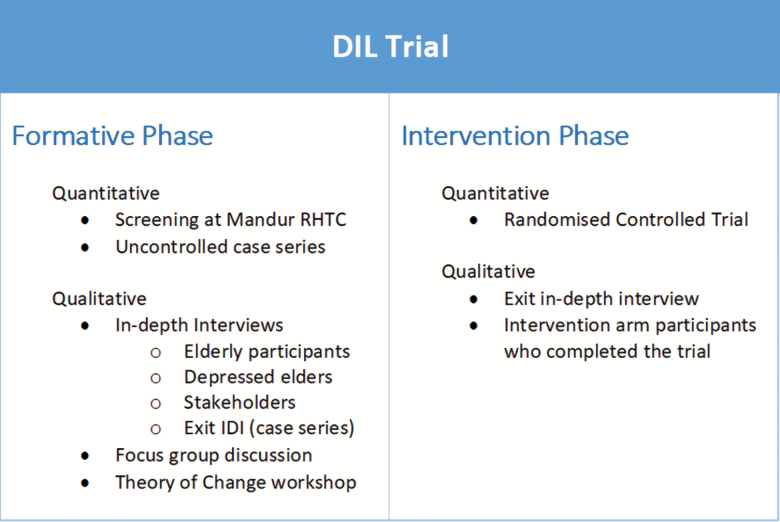
Summary of steps in DIL intervention development. “DIL intervention development” entailed a mixed-methods approach, with both qualitative and quantitative components.

DIL has followed the Medical Research Guidelines for developing and evaluating complex interventions (17). These guidelines emphasize (a) the importance of a sound theoretical understanding of how interventions cause change (ie, protect from depression, in the case of prevention), (b) that lack of effect may reflect implementation failure rather than genuine ineffectiveness, thus underscoring the need for a thorough evaluation process to identify implementation problems, and (c) the importance of adequate sample sizes to take account of variability of outcome, a range of outcomes to optimize use of data, and adaptation to local settings. We have adhered to MRC guidelines in the formative and pilot-intervention phases of DIL, guided by the work of Patel and colleagues (5) and Chatterjee and colleagues (18) in Goa. Figure 1 summarizes the steps accomplished in the formative and intervention phases of DIL. The use of focus groups with key stakeholders and theory of change workshops allowed us to form a logic model for the DIL intervention research, specifying stakeholders’ understanding of the current situation, the changes they hope to bring about through the program with and for whom, the activities planned to contribute toward this change, the resources needed to put into the effort, assumptions they are making, and external events that could influence results, needs of key stakeholders, resources available, acceptable methods of implementation, formative, and summative outcomes.

## Qualitative and Quantitative Results of the Formative Pilot Study of DIL

We enrolled 21 participants into DIL’s formative pilot study, aged 60 or older with subsyndromal symptoms of depression and anxiety as indicated by a score of 4 or greater on the General Health Questionnaire (GHQ) (19), but with no evidence of dementia as indicated by as score of 24 or higher on the Hindi Mini-Mental State Examination (20). Participants had high rates of medical polymorbidity, such as hypertension, diabetes, and painful osteoarthritis, typical of older adults in primary care settings. LHCs administered the DIL intervention, a mixture of PST-PC and BBTI, over 5–8 sessions, generally 30 minutes in length. As noted previously, PST-PC is behaviorally activating, teaching and reinforcing active coping skills and restoring a sense of self-efficacy (or confidence in one’s problem-solving ability). As illustrated by Figure 3, the first step in PST-PC involves defining a problem and goals to work toward. BBTI modifies an important risk factor for depression (ie, insomnia). Fortnightly conference calls with PST-PC and BBTI supervisors monitored delivery of DIL to maintain fidelity to the intervention models and also to allow adaptation to maximize acceptability and feasibility.

**Figure 2.**
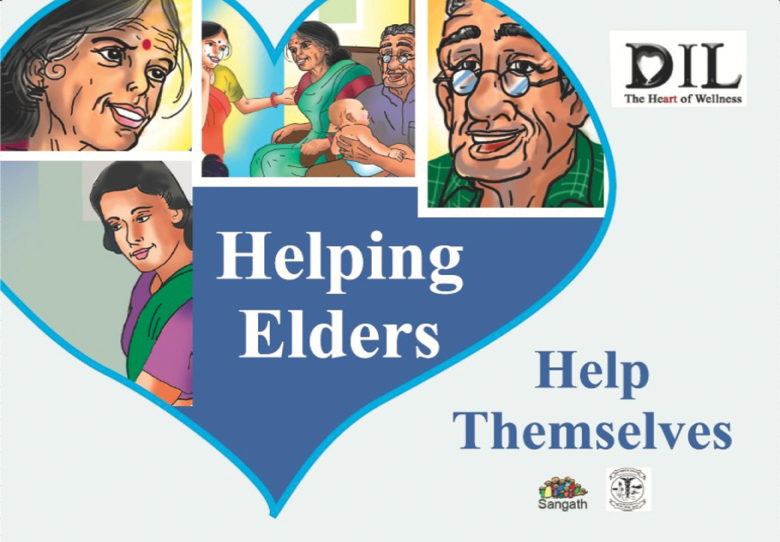
“Helping Elders Help Themselves.” The lay health counselor helps DIL participants choose a problem in their everyday lives that is causing “tension” or “worry,” then helps them brainstorm possible solutions and devise an action plan.

**Figure 3.**
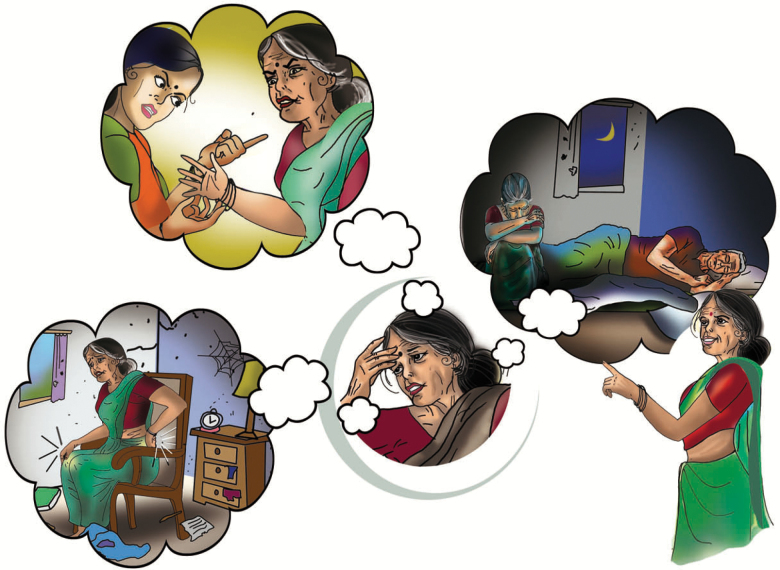
“Identifying the Problem” using PST. The lay health counselor teaches DIL participants the steps of problem solving therapy to improve active coping and self-confidence.

**Figure 4.**
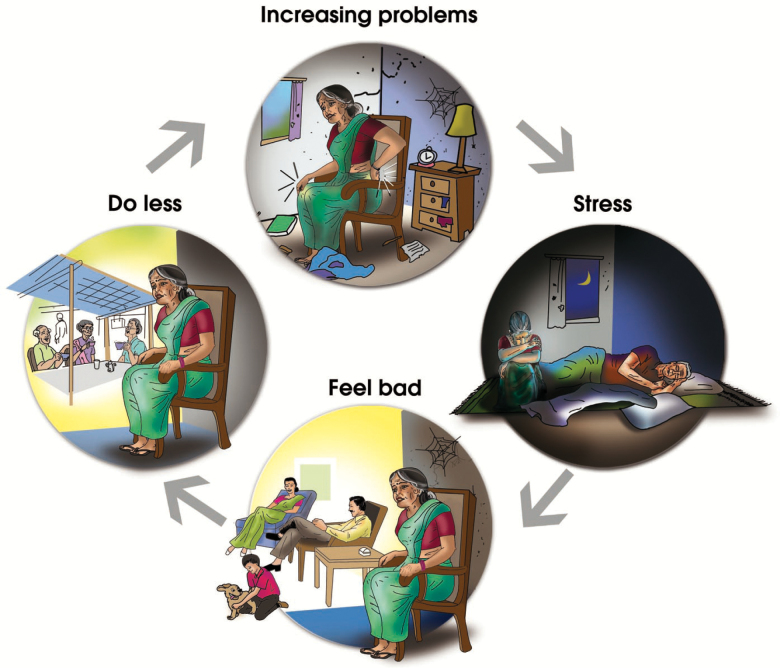
The Stress Cycle. The lay health counselor demonstrates how the negative effects of stress are made worse by ineffective coping and lack of activity.

Because of the low levels of literacy among DIL participants, we found it essential to use flip charts illustrations, as shown in Figures 2–4 and in the Supplementary Figures 1–6. The illustrations helped the participants grasp the strategies of PST-PC and BBTI, and put them to use in their daily lives. Examples of these “teaching” illustrations are found below in Figures 2–4. Other figures, included in Supplemental Material, illustrate teaching tools such as “Upward Spiral,” “Brief Behavioral Treatment for Insomnia,” “Mood Rating Scale,” and “Early Warning Signs of Diabetes.”

*Quantitative* results were available from 19 of the 21 participants initially enrolled, demonstrating a high level of retention (Table 1). Baseline mean score on the GHQ was 5.3 (standard deviation [*SD*]: 1.5). The postintervention GHQ score was 3.3 (*SD*: 2.0); paired *t* = 4.7, *df* = 18, *p* < .05.

**Table 1.** Demographic and Clinical Characteristics of Participants in Pilot Feasibility Study

Variable	No.	%
Gender		
Male	2	9.5
Female	19	90.5
Religion		
Hindu	20	95.2
Catholic	1	4.8
Education		
Illiterate	12	57.1
Literate	5	23.8
Up to primary school	4	19
Current living situation		
With son, daughter, or daughter-in-law	16	76.2
Separate from children	5	23.8
With spouse	3	14.3
Without spouse	18	85.7
Current medical condition		
Diabetes	6	28.6
Hypertension	13	61.9
Heart disease	5	23.8
High cholesterol	4	19
Stroke	1	4.8
COPD	3	14.3
Asthma	2	9.5
Arthritis	5	23.8
TB	0	0

Note: COPD = chronic obstructive pulmonary disease; TB = tuberculosis.

*Qualitative* analyses of in-depth interviews with participants showed that participants preferred the use of “tension” rather than “depression.” Many endorsed worries regarding an uncertain future related to relationships with their grown children and to deteriorating health (21). Participants endorsed the sessions as enjoyable.

## Architecture of the Final DIL Model as Tested in the Intervention Phase: A Randomized Indicated Prevention Trial Enrolling 181 Older Adults

As a result of our formative pilot work, the final DIL model comprised a hybrid of PST-PC and BBTI with education in self-management of chronic diseases like diabetes and with simple case management services (eg, pragmatic assistance with keeping medical appointments and treatment adherence). Because of the broad spectrum of risk for depression and anxiety disorders, interventions need to allow for some degree of tailoring to meet the specific needs of the individual. Thus, DIL enacts a structured but tailored approach that are responsive to individual needs and preferences.

**Figure 5.**
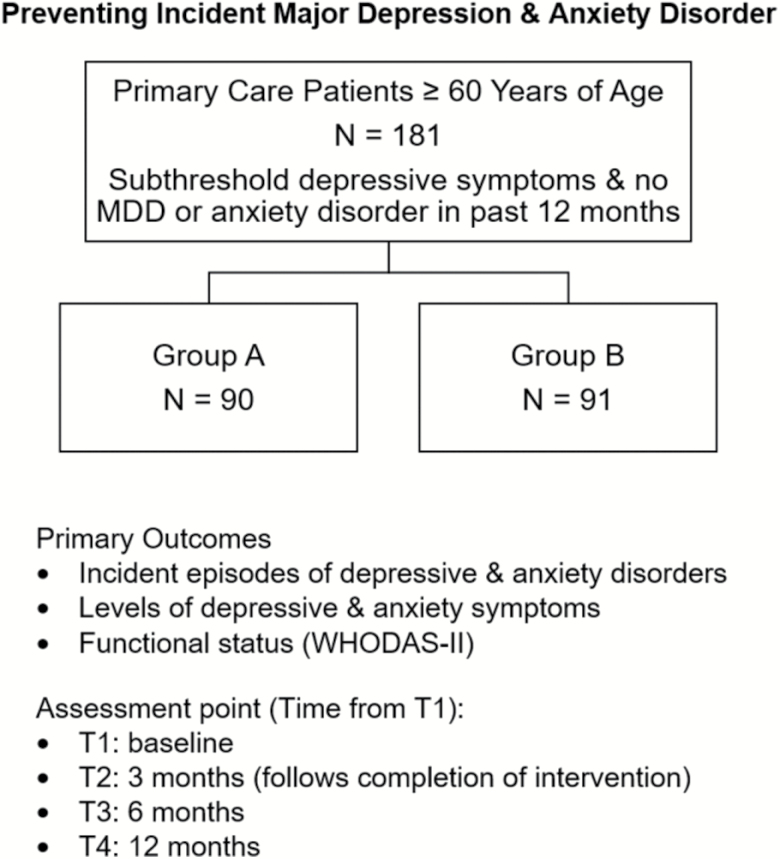
Phase II: pilot randomized prevention trial in 181 older adults.

The design of the ongoing randomized trial testing indicated depression prevention is depicted in Figure 5 below.

Our primary outcomes are both symptomatic burden (as reflected in GHQ scores) and the cumulative incidence of episodes of major depression and anxiety disorders over 12 months in the two arms of the study: “DIL” and enhanced usual care. We are tracking both depression and anxiety episodes because of their frequent co-occurrence as common mental health disorders in primary care. We are also tracking functional status via the WHODAS-II. Repeated assessments are conducted at baseline, upon completion of the DIL intervention, and at 6 and 12 months.

For ethical reasons related to the protection of human subjects, results of repeated assessments of mood and anxiety symptoms among DIL participants randomly assigned to enhance care as usual are made known to participants and their physicians on an ongoing basis. While enhancing care as usual in this fashion may make it more challenging to demonstrate meaningful differences between the intervention (DIL) and control arms (usual care), we believe that it is ethically mandatory to share such information. Thus, by monitoring all participants, regardless of randomized intervention assignment, we are able to identify early on those with need for clinical care for emerging depression, suicidal ideation, anxiety disorders, or dementia. We document the content of enhanced care as usual, that is, the extent to which participants receive either psychosocial or pharmacologic interventions for depression or anxiety.

Data analyses encompass both descriptive and inferential modeling. We are examining data descriptively using cross-tabulations, histograms, and tests for normality. Imputation strategies based in random regression models handle missing data. Time to onset of major depression or anxiety disorders as an outcome are modeled via survival analysis.

To date, we have demonstrated acceptable recruitment feasibility by meeting 100% of the targeted randomization (*n* = 181), with fewer than 20% of eligible subjects refusing randomization. Retention is also high, better than 85%. Randomization (via permuted block) has also been shown to be adequate by producing equal numbers of participants assigned to each condition, and having similar sociodemographic and clinical characteristics (Table 2).

**Table 2.** Demographic and Clinical Characteristics of Participants in “DIL” Randomized Clinical Trial

Variable	Mean		
	Participants = 181	Group A = 90	Group B = 91
Age	69.64	69.67	69.60
HMMSE	27.83	27.77	27.90
WHODAS	17.38	17.41	17.35
GHQ total	6.26	6.29	6.23
Gender			
Male	67	33	34
Female	114	57	57
Catchment area			
Rural (total)	123	63	63
Rural PHC	46	23	23
Rural community	80	40	40
Urban (total)	55	27	28
Urban PHC	18	9	9
Urban community	37	18	19
Self-reported chronic disease			
Diabetes mellitus	86	45	41
Hypertension	132	68	64
Heart disease	39	18	21
Stroke	15	8	7
Kidney disease	7	4	3
COPD	10	6	4
Asthma	11	4	7
Arthritis	70	39	31
Tuberculosis	7	3	4
Cancer	2	2	0
Mental illness^a^	7	4	3
Other^b^	14	9	5

Note: COPD = chronic obstructive pulmonary disease; GHQ = General Health Questionnaire; PHC = Primary Healthcare Clinic.

a Past mental illness (depression, anxiety).

b bpast miscellaneous medical illnesses.

## Summary DIL Translational Impact

DIL aims to generate the first evidence of potentially scalable interventions for prevention of depression and anxiety in later life from a LMIC witnessing rapid demographic transitions (Table 3).

**Table 3.** Key Aspects of Translational Impact

• Novel focus on prevention of common mental disorder in later life and in LMICs
• Use of lay health counselors to support scalability via use of task shifting and sharing
• Use of a combination of synergistic approaches encompassing problem solving training, brief behavioral treatment for insomnia, education in chronic disease self-management, and pragmatic case management to facilitate access to needed resources

Note: LMIC = low- and middle- income countries.

## Directions and Focus of Future Mental Illness Prevention Research in LMICs

We envision three key foci for future research: (a) identification of bio- and psychosocial risk markers, (b) expansion of outcomes to include measures of physical and neurological health status, and (c) enhancement of external validity and generalizability using large cluster-randomized trials to capture greater diversity in populations of interest. Markers of biological and psychosocial risk may provide important clues as to which persons, in particular, may benefit from the use of prevention strategies, thereby enabling more rational and efficient use of scare mental health resources in LMICs. Biomarkers could also have relevance to the development of staging models of depressive illness, thereby providing clues as to which type of intervention platform could be helpful to a given patient. In other words, such markers may also serve as moderators of intervention response, and they may tell us something about how interventions work to reduce the risk for common mental disorders. Because depression and anxiety do not occur in “pure” culture but rather co-occur with, and exacerbate, medical polymorbidity, mental illness depression research should also examine measures of medical and neurologic burden over time. It may be that by preventing depression, general measures of medical health will improve also and show lesser rates of incident disability. As such, depression prevention may be a key strategy to preserving the functional independence of older adults for as long as possible.

## Supplementary Material

Supplementary data are available at *Innovation in Aging* online.

igx030_suppl_Supplementary_Figures

## Funding

This work was supported by US National Institute of Mental Health (P30 MH90333, R34 MH96997), the Brain and Behavior Research Foundation, and the UPMC Endowment in Geriatric Psychiatry.

## Conflict of Interest

None reported.

## References

[1] Dias A, Azariah F, Cohen A, et al. Intervention development for the indicated prevention of depression in later life: the “DIL” protocol in Goa, India. Contemp Clin Trials Commun. 2017;6:131–139. 29057368 10.1016/j.conctc.2017.04.006 PMC5647889

[2] Reynolds CF, Thomas SB, Morse JQ, et al. Early intervention to preempt major depression among older black and white adults. Psychiat Serv. 2014;65(6):765–773. 10.1176/appi.ps.201300216 PMC4050338 24632760

[3] Rajkumar A, Thangadurai P, Senthilkumar P, Gayathri K, Prince M, Jacob K Nature, prevalence and factors associated with depression among the elderly in a rural south Indian community. Int Psychogeriatr. 2009;21(2):372–378. 19243657 10.1017/S1041610209008527 PMC3100909

[4] van Zoonen K, Buntrock C, Ebert DD, et al. Preventing the onset of major depressive disorder: a meta-analytic review of psychological interventions. Int J Epidemiol. 2014;43(2):318–329. 24760873 10.1093/ije/dyt175 PMC4023317

[5] Patel V, Weiss HA, Chowdhary N, et al. Effectiveness of an intervention led by lay health counsellors for depressive and anxiety disorders in primary care in Goa, India (MANAS): a cluster randomised controlled trial. Lancet. 2010;376(9758):2086–2095. 21159375 10.1016/S0140-6736(10)61508-5 PMC4964905

[6] Dobson KS. Handbook of Cognitive-Behavioral Therapies. New York, NY: Guilford Press; 2009.

[7] Buysse DJ, Germain A, Moul DE, et al. Efficacy of brief behavioral treatment for chronic insomnia in older adults. Arch Intern Med. 2011;171(10):887–895. 21263078 10.1001/archinternmed.2010.535 PMC3101289

[8] Barnes DE, Yaffe K The projected impact of risk factor reduction on Alzheimer’s disease prevalence. Lancet Neurol. 2011;10(9):819–828. 21775213 10.1016/S1474-4422(11)70072-2 PMC3647614

[9] Smit F, Ederveen A, Cuijpers P, Deeg D, Beekman A Opportunities for cost-effective prevention of late-life depression: an epidemiological approach. Arch Gen Psychiatry. 2006;63(3):290–296. 16520434 10.1001/archpsyc.63.3.290

[10] Robinson RG, Jorge RE, Moser DJ, et al. Escitalopram and problem-solving therapy for prevention of poststroke depression: a randomized controlled trial. JAMA. 2008;299(20):2391–400. 18505948 10.1001/jama.299.20.2391 PMC2743160

[11] Rovner BW, Casten RJ, Hegel MT, Leiby BE, Tasman WS Preventing depression in age-related macular degeneration. Arch Gen Psychiatry. 2007;64(8):886–892. 17679633 10.1001/archpsyc.64.8.886

[12] Van’t Veer-Tazelaar P, Smit F, van Hout H, et al. Cost-effectiveness of a stepped care intervention to prevent depression and anxiety in late life: randomised trial. Br J Psychiatry. 2010;196(4):319–325. 20357310 10.1192/bjp.bp.109.069617

[13] Fournier JC, DeRubeis RJ, Hollon SD, et al. Antidepressant drug effects and depression severity: a patient-level meta-analysis. JAMA. 2010;303(1):47–53. 20051569 10.1001/jama.2009.1943 PMC3712503

[14] Ciechanowski P, Wagner E, Schmaling K, et al. Community-integrated home-based depression treatment in older adults: a randomized controlled trial. JAMA. 2004;291(13):1569–1577. 15069044 10.1001/jama.291.13.1569

[15] Reynolds CF III, Cuijpers P, Patel V, et al. Early intervention to reduce the global health and economic burden of major depression in older adults. Annu Rev Public Health. 2012;33:123–135. 22429161 10.1146/annurev-publhealth-031811-124544 PMC3356692

[16] Baldwin RC Preventing late-life depression: a clinical update. Int Psychogeriatr. 2010;22(8):1216–1224. 20594389 10.1017/S1041610210000864

[17] Craig P, Dieppe P, Macintyre S, Michie S, Nazareth I, Petticrew M Developing and evaluating complex interventions: the new Medical Research Council guidance. BMJ. 2008;337:a1655. 18824488 10.1136/bmj.a1655 PMC2769032

[18] Chatterjee S, Chowdhary N, Pednekar S, et al. Integrating evidence-based treatments for common mental disorders in routine primary care: feasibility and acceptability of the MANAS intervention in Goa, India. World Psychiatry. 2008;7(1):39–46. 18458786 10.1002/j.2051-5545.2008.tb00151.x PMC2359726

[19] Goldberg D. General Health Questionnaire (GHQ-12).Windsor, UK: Nfer-Nelson; 1992.

[20] Ganguli M, Ratcliff G, Chandra V, et al. A Hindi version of the MMSE: the development of a cognitive screening instrument for a largely illiterate rural elderly population in India. Int J Geriatr Psychiatry. 1995;10(5):367–377.

[21] Cohen A, Dias A, Azariah F, et al. Aging and well-being in Goa, India: a qualitative study. Aging Ment Health. 2016;22:1–7. 27689842 10.1080/13607863.2016.1236239 PMC5374050

