# Supplementary material for: Preventing Late-Life Depression: Lessons in Intervention Development From Goa, India
Source: Innov Aging. 2018 Jan 24;1(3):igx030. doi: 10.1093/geroni/igx030 (PMC6243671; doi:10.1093/geroni/igx030)
Supplement: igx030_suppl_Supplementary_Figures [file igx030_suppl_supplementary_figures.docx]

Supplementary Figure 1: "Problem Solving Therapy” Upward Spiral


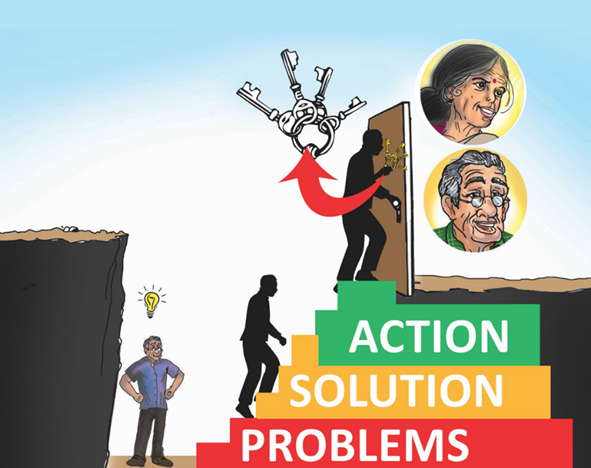


The lay health counsellor teaches DIL participants how to better define problems, set realistic goals, test and evaluate strategies.   This helps DIL participants have a sense of self-confidence in the face of difficult challenges that can produce "tension" or "worry".

Supplementary Figure 2: “Brief Behavioral Treatment for Insomnia” Do not lie in bed for more than 30 minutes.


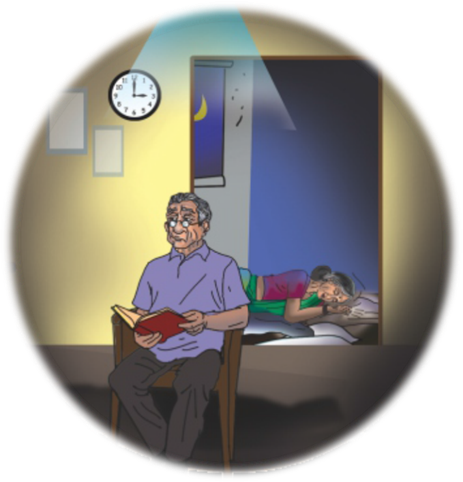


The lay health counsellor teaches DIL participants the basic strategies of BBTI, such as a regular bedtime and getting up time, not staying in bed unless sleepy, and not returning to bed unless sleepy.   BBTI addresses the hyperarousal of insomnia disorder.

Supplementary Figure 3: Sleep-Wake Routine


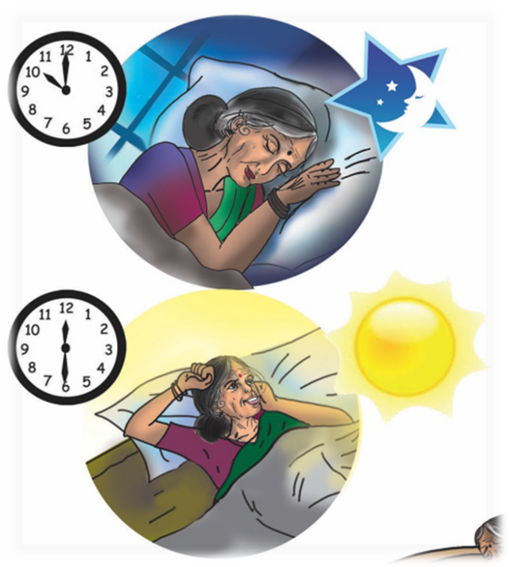


The Lay Health Counselor teaches DIL participants the importance of establishing and sticking with a regular bedtime and rise time, to improve sleep quality.

Supplementary Figure 4: Mood Rating Scale


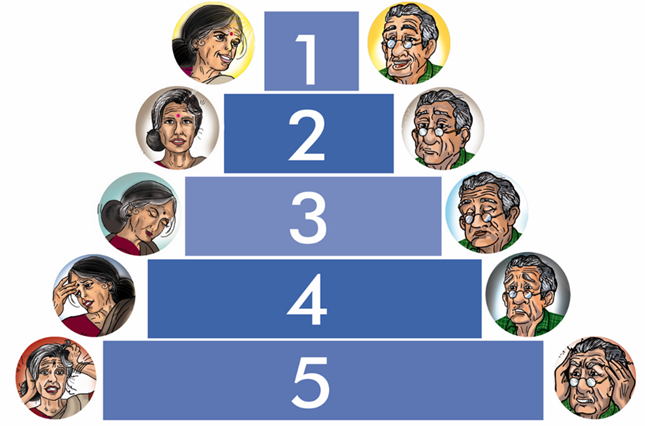


The lay health counsellor uses the mood rating scale as a tool to teach DIL participants how to gauge their mood and overall feeling state since the last visit.

Supplementary Figure 5: Identifying healthy vs. non-healthy strategies


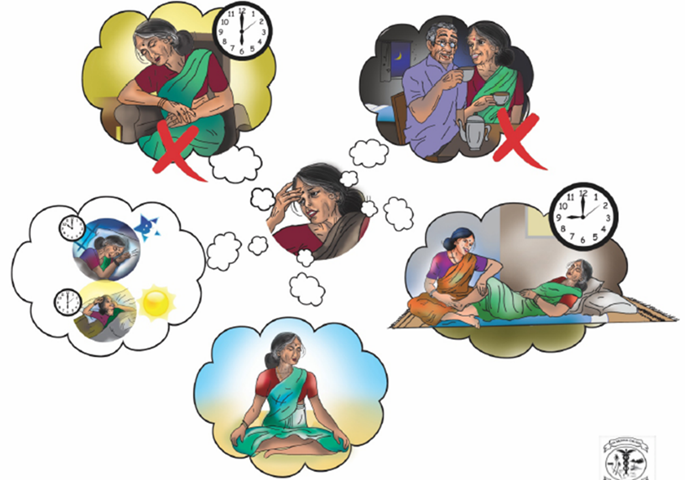


The Lay Health Counsellor teaches DIL participants about habits that interfere with sleep, such as drinking caffeinated beverages or worrying and ruminating while in bed.

Supplementary Figure 6: "Early Warning Signs of Diabetes “


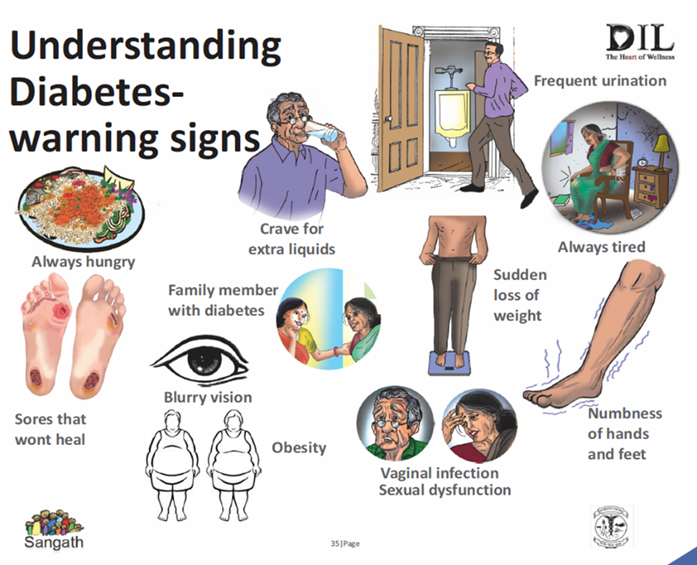


The Lay Health Counselor teaches DIL participants about better self-care for living with diabetes.
